# The presence of human mesenchymal stem cells of renal origin in amniotic fluid increases with gestational time

**DOI:** 10.1186/s13287-018-0864-7

**Published:** 2018-04-25

**Authors:** Md Shaifur Rahman, Lucas-Sebastian Spitzhorn, Wasco Wruck, Carsten Hagenbeck, Percy Balan, Nina Graffmann, Martina Bohndorf, Audrey Ncube, Pascale V. Guillot, Tanja Fehm, James Adjaye

**Affiliations:** 1 Institute for Stem Cell Research and Regenerative Medicine, Medical Faculty, Heinrich Heine University, Moorenstraße 5, 40225 Düsseldorf, Germany; 2 Department of Obstetrics and Gynaecology, Medical Faculty, Heinrich Heine University Düsseldorf, Moorenstraße 5, 40225 Düsseldorf, Germany; 3 Institute for Women’s Health, Maternal and Fetal Medicine Department, University College London, London, WC1E 6HX UK

**Keywords:** Amniotic fluid, Kidney, Renal progenitor cells, SIX2, Mesenchymal stem cells, Albumin endocytosis, Third trimester

## Abstract

**Background:**

Established therapies for managing kidney dysfunction such as kidney dialysis and transplantation are limited due to the shortage of compatible donated organs and high costs. Stem cell-based therapies are currently under investigation as an alternative treatment option. As amniotic fluid is composed of fetal urine harboring mesenchymal stem cells (AF-MSCs), we hypothesized that third-trimester amniotic fluid could be a novel source of renal progenitor and differentiated cells.

**Methods:**

Human third-trimester amniotic fluid cells (AFCs) were isolated and cultured in distinct media. These cells were characterized as renal progenitor cells with respect to cell morphology, cell surface marker expression, transcriptome and differentiation into chondrocytes, osteoblasts and adipocytes. To test for renal function, a comparative albumin endocytosis assay was performed using AF-MSCs and commercially available renal cells derived from kidney biopsies. Comparative transcriptome analyses of first, second and third trimester-derived AF-MSCs were conducted to monitor expression of renal-related genes.

**Results:**

Regardless of the media used, AFCs showed expression of pluripotency-associated markers such as SSEA4, TRA-1-60, TRA-1-81 and C-Kit. They also express the mesenchymal marker Vimentin. Immunophenotyping confirmed that third-trimester AFCs are bona fide MSCs. AF-MSCs expressed the master renal progenitor markers SIX2 and CITED1, in addition to typical renal proteins such as PODXL, LHX1, BRN1 and PAX8. Albumin endocytosis assays demonstrated the functionality of AF-MSCs as renal cells. Additionally, upregulated expression of *BMP7* and downregulation of *WT1*, *CD133*, *SIX2* and C-Kit were observed upon activation of WNT signaling by treatment with the GSK-3 inhibitor CHIR99201. Transcriptome analysis and semiquantitative PCR revealed increasing expression levels of renal-specific genes (e.g., *SALL1*, *HNF4B*, *SIX2*) with gestational time. Moreover, AF-MSCs shared more genes with human kidney cells than with native MSCs and gene ontology terms revealed involvement of biological processes associated with kidney morphogenesis.

**Conclusions:**

Third-trimester amniotic fluid contains AF-MSCs of renal origin and this novel source of kidney progenitors may have enormous future potentials for disease modeling, renal repair and drug screening.

**Electronic supplementary material:**

The online version of this article (10.1186/s13287-018-0864-7) contains supplementary material, which is available to authorized users.

## Background

A functional kidney is essential for healthy living due to its major role in toxin and drug filtration. Globally, each year millions of patients require rapid kidney transplantation or dialysis to restore renal function [1, 2]. But the shortage of compatible organs, donor-associated diseases, ageing-associated factors and high cost of transplantation/dialysis are major hurdles [3]. Kidney-associated dysfunctions are now a prioritized health concern and research area. A potential alternative source of renal cells are those derived from embryonic and induced pluripotent stem cells (ESCs and iPSCs) [4–9]. Clinical applications of pluripotent stem cell technologies are constrained by the risk of tumor formation, immunological rejection, legal as well as ethical concerns. In light of this, it is therefore important to find other sources of stem cells which are not tumorigenic, bear a broad differentiation potential and have a high renal regenerative potential.

The architecture and organization of the kidney is very complex and consists of numerous cell types [10] which can interchange identities by a very complex reciprocal interplay and interactions of stromal and epithelial cell lineages [9, 11]. Kidney mesenchymal cells have been demonstrated to express SIX2 and Cbp/p300-interacting transactivator 1 (*CITED1*) which are crucial for the self-renewing capability [11, 12]. Furthermore, the main kidney nephron-regulatory genes have been described, such as SALL1, PAX2, WT1, Cytokeratin 19 (CK19), CD133, Podocalyxin-like protein 1 (*PODXL*), *HOXD11*, *HNF1B*, BRN1, Lhx1 and Pax8 [13–18]. Adult and fetal bone marrow-derived mesenchymal stem cells (BM-MSCs) have been shown to be capable of repairing renal function deficits [19, 20]. BM-MSCs have potent immunosuppressive properties and their potential application in acute kidney injury animal models has been studied recently [21]. However, the use of adult BM-MSCs has some limitations such as the low number of MSCs in adult bone marrow, expression of ageing-associated factors, slow expansion rate, early senescence, inactive telomerase, shorter telomeres and restricted differentiation potential [22]. Due to the increasing number of patients with kidney diseases and limited cell-based therapeutic options, alternative renal progenitor cells and sources are clearly in need [23]. Amniotic fluid contains fetal-derived differentiated and undifferentiated progenitor cells. In vitro, they can be expanded in distinct media formulations and exhibit a heterogeneous morphology with a preponderance of epithelioid and fibroblastoid mesenchymal-like cell shape [24]. In 2007, Perin et al. [25] demonstrated the potency of second-trimester amniotic fluid-derived MSCs (AF-MSCs) to form embryonic kidney structures in vitro. Later, they also showed that human AF-MSCs help in regenerating kidneys undergoing acute tubular necrosis in a rodent model [26–28]. In an animal model of acute renal injury, Camussi’s research group confirmed these results and could show comparable efficacy between BM-MSCs and AF-MSCs [29]. Remarkably, the renal differentiation potential of AF-MSCs was demonstrated by producing chimeric organotypic renal structures from murine embryonic kidney cells and human AF-MSCs [30]. Although properties of human amniotic fluid cells such as the lack of immunogenicity and tumorigenicity, their anti-inflammatory properties and their high proliferative and differentiation potential are well described [31, 32], the exact origin of AF-MSCs is still unknown and controversial [33]. Before use in clinical applications, it is mandatory to elucidate the origin of AF-MSCs. Due to the fact that term amniotic fluid consists mostly of fetal urine [34], we hypothesized that AF-MSCs originate from the kidney and accumulate in the AF during fetal nephrogenesis.

Adult and neonatal human urine has been described as a source of kidney progenitor cells [35, 36]. Second-trimester AFCs have been described as an alternative source of podocytes [37] which express mesenchymal markers as well as the podocyte markers CD2AP and NPHS2 [25, 38]. In this study we isolated third-trimester human AF-MSCs and cultured them in distinct supporting media. AF-MSCs were characterized as a multipotent population of renal cells. By combining cellular, molecular, functional and transcriptome data, we conclude that third-trimester amniotic fluid harbors MSCs originating from the fetal kidney. These cells should be considered promising sources for studies on kidney development, nephrotoxicity tests, disease modeling, drug screening and future kidney-related cellular therapies.

## Methods

### Isolation and culture of human amniotic fluidic cells

Healthy donors who provided the first and second-trimester amniotic fluid in this study provided written informed consent in accordance with the Declaration of Helsinki. Ethical approval was given by the Research Ethics Committees of Hammersmith & Queen Charlotte’s Hospitals (2001/6234) in compliance with UK national guidelines (Review of the Guidance on the Research Use of Fetuses and Fetal Material (1989), also known as the Polkinghorne Guidelines, Her Majesty’s Stationery Office, London, 1989: Cm762) for the collection of fetal tissue for research. Third-trimester amniotic fluid samples from healthy donors were collected from the Department of Obstetrics and Gynaecology, Medical Faculty, Heinrich Heine University Düsseldorf, Germany, with informed patient consent as well as institutional ethical approval. Amniotic fluids were processed and AF-MSCs were isolated as described previously [32]. In brief, the cells were cultured in Prime XV or Chang C Medium (both Irvine Scientific, CA, USA), αMEM (Minimum Essential Medium Eagle Alpha Modification; Sigma) containing 10% FBS, 1% GlutaMAX and 1% penicillin/streptomycin (Penstrep), MG30 (Cell Lines Service, Germany) or renal cell medium (RCM) consisting of high-glucose Dulbecco’s Modified Eagle’s Medium (DMEM) supplemented with 1% Penstrep, 1% glutamine, 10% FBS and SingleQuot Kit CC-4127 REGM at 37 °C, 5% CO_2_ and 5% O_2_ (Lonza) [39, 40].

After the appearance of initially attached cells (days 4–7), the medium was changed and cells grew until reaching almost full confluency. The cells were then detached using TrypLE Express (Thermo Fisher Scientific) and prepared for further passaging and experiments. Urine-derived kidney progenitor cells and corresponding iPSCs (UM51, ISRM-UM51 [40]) and human renal epithelial cells from a biopsy (HREpCs; PromoCell, Heidelberg, Germany) were used as control cells.

### Immunofluorescence staining

To analyze the cells for pluripotency, mesenchymal stem cell and renal cell specific markers, AF-MSCs were cultured in 12-well plates. After a washing step using PBS (Gibco), 4% paraformaldehyde (PFA; Polysciences Inc., PA, USA) was used to fix the cells for 15 min at room temperature (RT). To increase the cell membranes’ permeability, 1% Triton X-100 (Carl Roth GmbH & Co. KG, Karlsruhe, Germany) was applied to the fixed cells for 5 min followed by blocking of unspecific binding sites for 2 h. For staining of intracellular proteins, this blocking buffer contained 10% normal goat serum (NGS; Sigma), 0.5% Triton X-100, 1% BSA (Sigma) and 0.05% Tween 20 (Sigma), all dissolved in PBS. Triton and Tween were omitted when extracellular proteins were stained. Afterward, the primary antibodies (presented in Additional file 1: Table S1) were diluted in blocking buffer/PBS and incubated with the cells for 1 h at RT followed by several washing steps using 0.05% Tween 20 in PBS. The corresponding secondary Cy3-labeled or Alexa Fluor 488-labeled antibodies (Thermo Fisher Scientific) and Hoechst 33,258 dye (Sigma-Aldrich Chemie GmbH, Taufkirchen, Germany) or DAPI (Southern Biotech) were added under light exclusion. For the actin filament staining, the toxin phalloidin 488 (A12370; Life Technologies) was used in a dilution of 1:200. A fluorescence microscope (LSM700; Zeiss, Oberkochen, Germany) was used for taking the pictures. All pictures were processed with the ZenBlue 2012 Software Version 1.1.2.0. (Carl Zeiss Microscopy GmbH, Jena, Germany).

### In-vitro differentiation assay

In-vitro differentiation of the AF-MSCs into adipocytes, chondrocytes and osteoblasts was done employing the StemPro Adipogenesis, Chondrogenesis, and Osteogenesis differentiation Kits (Gibco, Life Technologies, CA, USA). Media were replaced 2–3 times per week for 3 weeks, and the formation of intracellular lipid droplets (adipocytes), calcium mineralization (osteoblasts) and cellular aggregation toward clusters (chondrocytes) was observed from 14 to 21 days. After the differentiation process, fixation of the cells was done using 4% PFA for 30 min at RT. Subsequently, the cells were stained with Oil Red O for adipocytes, Alcian Blue for chondrocytes, and Alizarin Red S for osteoblasts as described previously [32]. A light microscope was used for imaging.

### Flow cytometric analysis

The human MSC phenotyping kit (Miltenyi Biotec GmbH, Bergisch Gladbach, Germany) was used to analyze the cell surface marker composition of the AF-MSC samples (2 × 10^5^ cells were used for each analysis), according to the manufacturer’s instructions. The cells were washed with PBS and centrifuged at 300 × *g* for 5 min. After resuspending the pellet in 100 μl PBS, 0.5 μl of the MSC phenotyping cocktail or of the isotype control cocktail were added and the tubes were mixed thoroughly. The MSC phenotyping cocktail is composed of a mixture of fluorochrome-coupled antibodies against various cell surface proteins (CD14-PerCP, CD20-PerCP, CD34-PerCP, CD45-PerCP, CD73-APC, CD90-FITC and CD105-PE). The isotype phenotyping cocktail served as a negative control. The antibody binding took place at 4 °C for 10 min in the dark. Nonbound antibodies were washed out using 1 ml PBS. After centrifugation at 300 × *g* for 5 min, cell fixation using 4% PFA was done.

To analyze the AF-MSCs for pluripotency-associated cell surface markers (TRA-1-60, TRA-1-81, stage-specific embryonic antigen 4 (SSEA4)), corresponding prelabeled antibodies (anti-TRA-1-60-PE, human (clone REA157), number 130-100-347; anti-TRA-1-81-PE, human (clone REA246), number 130-101-410, and anti-SSEA-4-PE, human (clone REA101), number 130-098-369; Miltenyi Biotec GmbH, Bergisch Gladbach, Germany) were used. The staining procedure was carried out as already described. Until analysis via BD FACSCanto (BD Biosciences, Heidelberg, Germany) and CyAn ADP (Beckman Coulter, CA, USA), stained cells were kept at 4 °C in the dark. The FCSalyzer software version 0.9.3 and Summit 4.3 software were used for data analysis.

### RNA isolation and quantitative PCR

After single washing with PBS, TRIzol (Thermo Fisher) was added to the cells for 5 min at RT, and the cells were scraped off and stored at − 80 °C. For isolation of the RNA, the Direct-zol RNA Miniprep Kit (Zymo Research, CA, USA) was used according to the manufacturer’s instructions. All of the primers used were purchased from MWG (primer sequences and predicted sizes of amplicons presented in Additional file 1: Table S2). After checking the quality of mRNA, complementary DNA (cDNA) was synthesized with the TaqMan Reverse Transcription Kit (Applied Biosystems). A sample of 500 ng of RNA was used for cDNA synthesis. The prepared mix of 20 μl per sample consisted of 7.70 μl H_2_O, 2 μl reverse transcriptase buffer, 4.4 μl MgCl_2_ (25 mM), 1 μl Oligo (dT)/random hexamer (50 μM), 4 μl dNTP mix (10 mM), 0.4 μl RNase inhibitor (20 U/μl) and 0.5 μl reverse transcriptase (50 U/μl). For semi-qPCR, a mixture of 25 μl per sample contained the following: 11.375 μl H_2_O, 5 μl of 1× Go-Taq G2 Hot Start Green PCR buffer, 4 μl of 4 mM MgCl_2_, 0.5 μl dNTP-Mix (10 mM each), 1 μl forward primer (0.3 μM), 1 μl reverse primer (0.3 μM), 0.125 μl (0.625 U) Hotstart Taq polymerase (5 U/μl) and 2 μl cDNA. A PCR thermal cycler (PEQLAB, Erlangen Germany) was employed. After an initial denaturation step at 95 °C for 2 min, 30 cycles followed with a denaturation step at 95 °C for 30 s, an annealing step at the temperature specific for each primer (ranging from 55 to 63 °C) for 30–35 s and an extension step at 72 °C for 30–40 s. Detection of semi-qPCR amplification products was performed by size fractionation on 2% agarose gel electrophoresis. Real-time quantitative PCR was performed in technical triplicates with Power SYBR Green Master Mix (Life Technologies) on a VIIA7 (Life Technologies) machine. Mean values were normalized to levels of the housekeeping gene ribosomal protein L37A. Results are depicted as mean values (% of untreated control) with 95% confidence interval.

### Albumin endocytosis assay

To analyze the functional ability of the AF-MSCs to endocytose exogenous albumin, cells were plated at a density of 30% in 12-well plates without coating. After 2 days, media were supplemented with 10 μM CHIR dissolved in DMSO or the same volume of DMSO alone and the cells were allowed to differentiate for 2 days. After this time period the cells were washed once with PBS and incubated in new medium supplemented with 20 μg/ml of albumin from bovine serum (BSA), Alexa Fluor™ 488 conjugate (catalog no. A13100; Thermo Fischer) for 1 h. As endocytosis is an energy-dependent process, incubations were performed at 37 °C. After 1 h of incubation, however, cells were washed three times with ice-cold PBS and fixed with 4% PFA for 15 min. Cell-associated fluorescence was analyzed using an excitation wavelength of 488 nm and an emission wavelength of 540 nm and imaged using a florescence microscope (LSM700; Zeiss, Oberkochen, Germany). All pictures were analyzed with ZenBlue 2012 Software Version 1.1.2.0. (Carl Zeiss Microscopy GmbH, Jena, Germany). Human fetal foreskin cell line HFF1 (SCRC-1041; ATCC) and fMSCs (kindly gifted from Prof. Richard O. C. Oreffo, Southampton, UK) served as control.

### Transcriptome analysis

PrimeView Human Gene Expression Array Chips (Affymetrix; Thermo Fisher Scientific) were used for microarray experiments (conducted by Biologisch-Medizinisches Forschungszentrum, Düsseldorf, Germany). The gene expression profile for AF-MSCs, HREpCs, fMSCs, UM51 and ISRM-UM51 are provided online at the National Center of Biotechnology Information Gene Expression Omnibus. The affy package of the R/Bioconductor environment [41, 42] was used for further processing of the unnormalized bead summary data. After background correction, the data were transformed to a logarithmic scale (to base 2), and normalized by employing the robust multiarray average method. The heatmap.2 function from the gplots package (http://CRAN.R-project.org/package=gplots) was employed to create cluster analysis and heatmaps. The correlation coefficients were calculated with Pearson correlation as a similarity measure (http://CRAN.R-project.org/package=gplots). Based on the results of the transcriptome analysis, the DAVID tool (https://david.ncifcrf.gov/) [43] was used to generate gene ontology terms and associated KEGG pathways [44] for the distinct gene sets.

## Results

### AFCs become homogeneous after several passages and contain a renal cell-like subpopulation

**Fig. 1:**
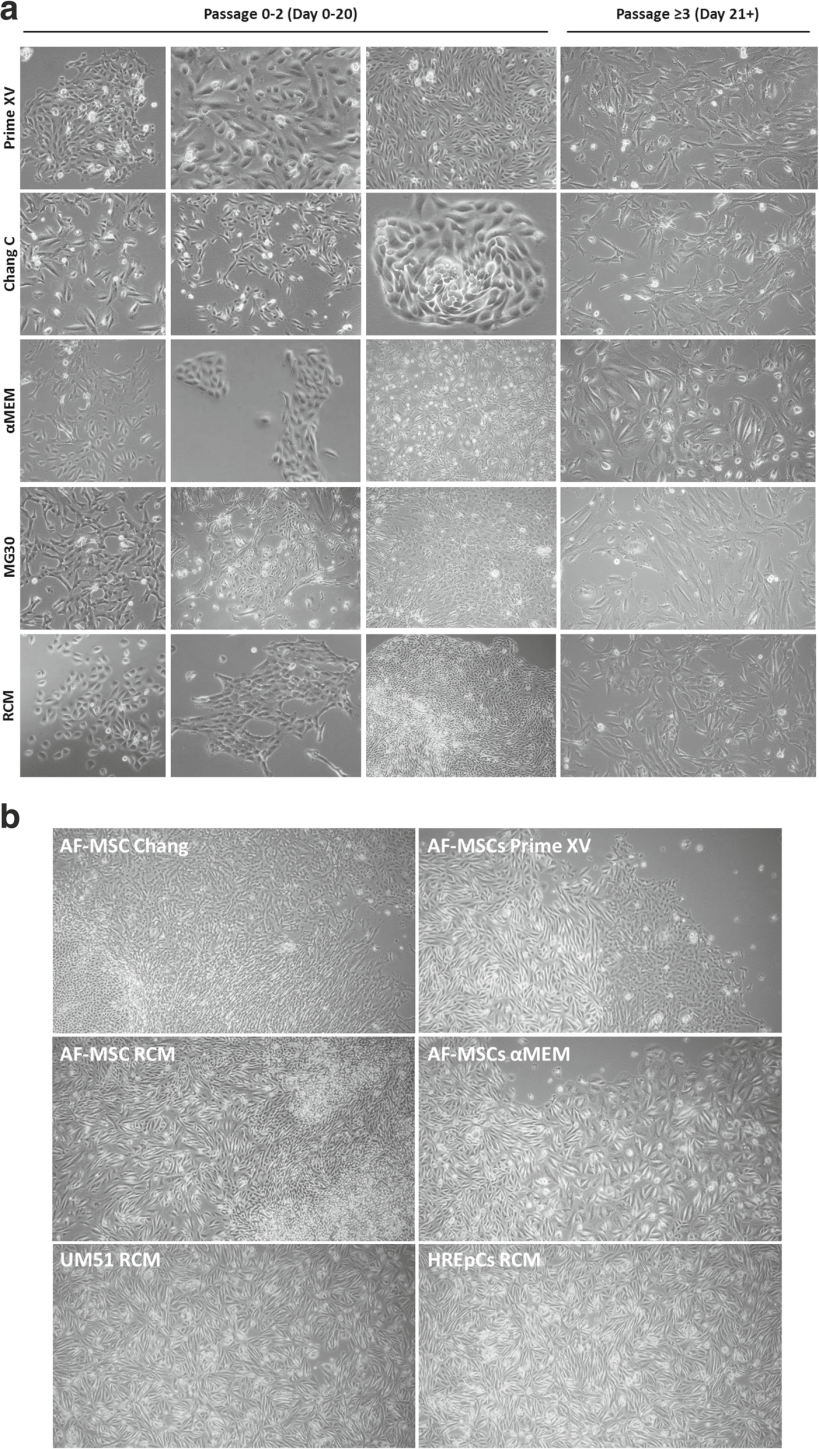
Initially attached heterogeneous AFCs display homogeneous cell morphology upon passaging and include typical kidney cell morphologies. Phenotypical complexity of AFCs observed, regardless of culture media composition within first two passages. Upon further passaging, cells became homogeneous with fibroblast-like morphology (**a**). During culture, subpopulation of cells observed which had similar morphology to distinct renal-originated progenitor cells UM51 and HREpCs (**b**). AF-MSC amniotic fluid mesenchymal stem cell, HREpC human renal epithelial cell, αMEM minimum essential medium alpha modification, RCM renal cell medium

In-vitro growth of AFCs in monolayers preserves diversity of cell types irrespective of media, in particular within the first two passages. AFCs were isolated and expanded in various media (RCM, Prime XV, Chang C, MG30, αMEM) under hypoxic conditions. Bright-field microscopic observation revealed a mixture of distinct cell types with a morphology of mesenchymal-like, epithelioid, spindle, cobble-stone and tubular shapes, of fetal-derived differentiated and undifferentiated progenitor cells (Fig. 1a). After passaging 3–4 times, the cells became more homogeneous with fibroblastic mesenchymal-like morphologies in all media except αMEM and MG30. In αMEM and MG30, cells attained an oval/egg-shaped morphology and had significantly decreased growth rates (Fig. 1a). Based on morphology, renal progenitor, undifferentiated and differentiated cell types were observed as subpopulations. The commercially available human kidney cell line HREpC as well as urine-derived renal cells (UM51) served as controls (Fig. 1b).

### AFCs express pluripotency-associated proteins

**Fig. 2:**
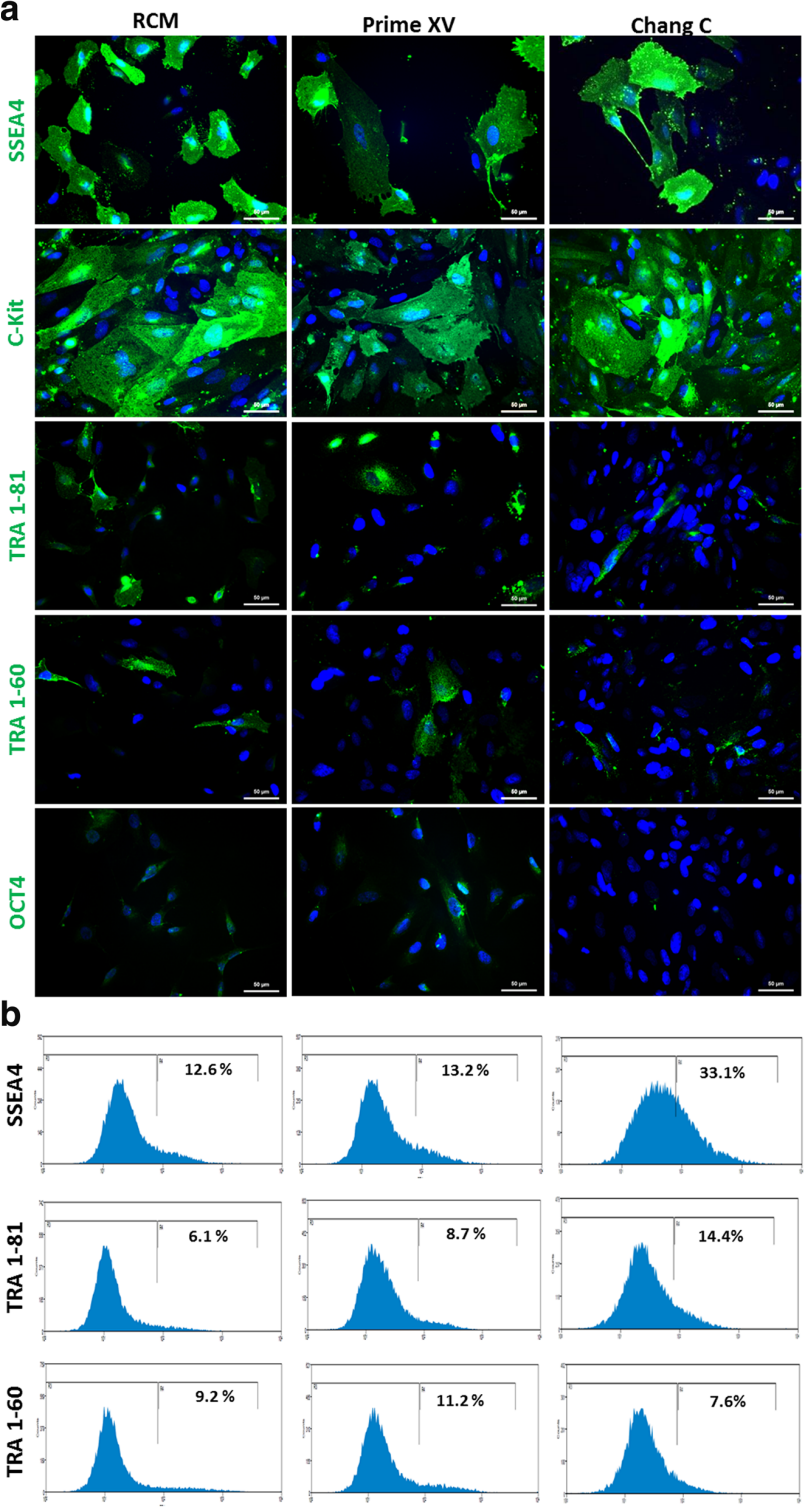
Pluripotency-associated stem cell marker expression of AFCs in distinct media. Immunofluorescent-based staining showed similar stem cell-related protein expression in all three media conditions (RCM, Prime XV, Chang C). AFCs express SSEA4, C-Kit, TRA-1-60 and TRA-1-81. Cell nuclei stained using Hoechst (**a**). Flow cytometric analysis confirmed cell surface expression of SSEA4, TRA-1-60 and TRA-1-81 (**b**). RCM renal cell medium

To analyze the presence of pluripotent stem cell-associated markers in AFCs, both immunofluorescence staining and flow cytometry were performed. The AFC populations cultured in RCM, Prime XV and Chang C media were found to express SSEA4, C-Kit, TRA-1-60 and TRA-1-81. Expression of cytoplasmic and no nuclear octamer-binding transcription factor 4 (OCT4) was observed at early passages in RCM and Prime XV (Fig. 2a). However, the percentages of cells positive for the investigated markers (SSEA4, C-Kit, TRA-1-60 and TRA-1-81) were consistent with the flow cytometric data. Approximately 13% of the RCM cultured cells were positive for SSEA4, 9% for TRA-1-60 and 6% for TRA-1-81. AFCs cultured in Prime XV showed positivity rates of 13.2% for SSEA4, 8.7% for TRA-1-60 and 11.2% for TRA-1-81. Previously performed flow cytometric analysis for cells cultured in Chang C media revealed 33.1% SSEA4, 14.4% TRA-1-60 and 7.6% TRA-1-81 positive cells [32] (Fig. 2b).

### AFCs express proteins related to MSCs as well as CK19 and show multilineage differentiation potential in vitro

**Fig. 3:**
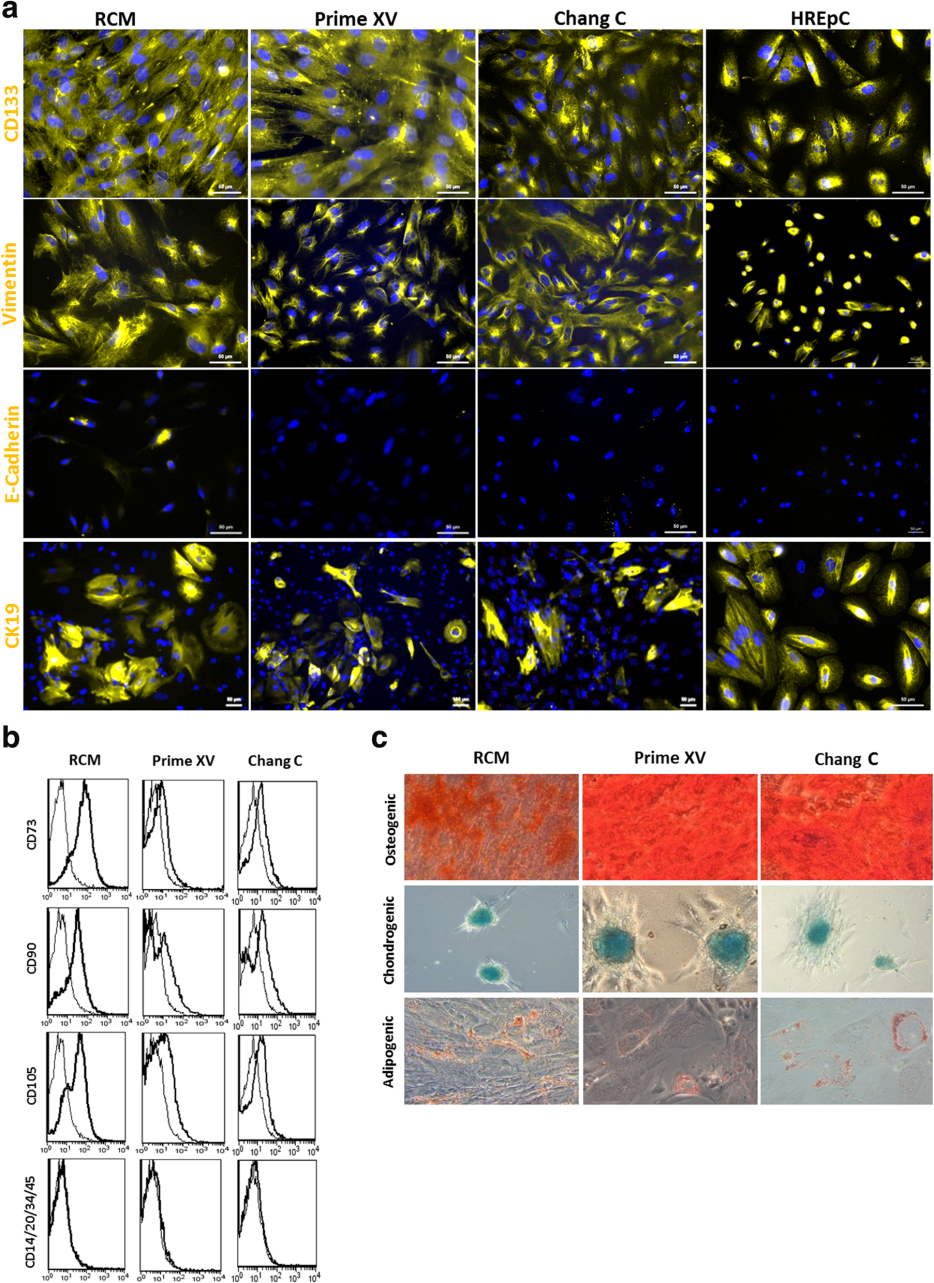
AFCs express typical MSC-associated proteins as well as CK19 and show multilineage differentiation potential in vitro. AFCs cultured in distinct media compositions stained positive for Vimentin and CD133 but negative for E-Cadherin. CK19, an established kidney epithelial marker, also expressed. Cell nuclei stained using Hoechst/DAPI (**a**). Flow cytometry-based analysis confirmed expression of MSC markers CD73, CD90 and CD105, and negativity for hematopoietic markers CD14, CD20, CD34 and CD45 (antibody isotype controls represented by thin lines; bold lines indicate histograms of distinct proteins) (**b**). Analysis of multilineage differentiation capacity of AF-MSCs revealed Alizarin Red staining of osteoid matrix-like structure in osteogenic medium, Alcian Blue staining of proteoglycans in chondrogenic media and lipid droplet formation around the cells in adipogenic media (**c**). HREpC human renal epithelial cell, RCM renal cell medium

AFCs cultured in all three media compositions (RCM, Prime XV and Chang C) expressed the typical mesenchymal marker Vimentin and not E-Cadherin. Additionally, subpopulations of the cells were positive for CK19, a marker for renal epithelial cells. The expression of CD133/Prominin-1, as a marker for multipotent progenitor cells, was also observed in all conditions. HREpCs, derived from kidney biopsies, were used as renal reference cells and also showed positivity for Vimentin, CD133 and CK19 (Fig. 3a). To analyze the typical MSC surface marker expression in AFCs, flow cytometric analysis was conducted. The presence of CD73, CD90 and CD105 could be identified in all cases; interestingly, cells in RCM had the highest level of expression. However, all cell preparations were devoid of the hematopoietic markers CD14, CD20, CD34 and CD45 (Fig. 3b, [32]). These features establish and confirm these cells as bona fide AF-MSCs.

To investigate the differentiation capacity of the AFCs, the cells were subjected to adipocyte, chondrocyte and osteoblast differentiation for 3 weeks. Successful differentiation into adipocytes was observed by Oil Red O staining of emerging fat droplets surrounding the cell nuclei. During chondrogenic differentiation the cells aggregated, and Alcian Blue staining showed the presence of emerged proteoglycans within the developed cell clusters. Osteogenic lineage differentiation was shown by Alizarin Red S staining of developed calcium deposits (Fig. 3c).

### AF-MSCs express renal markers irrespective of in-vitro culture media composition

**Fig. 4:**
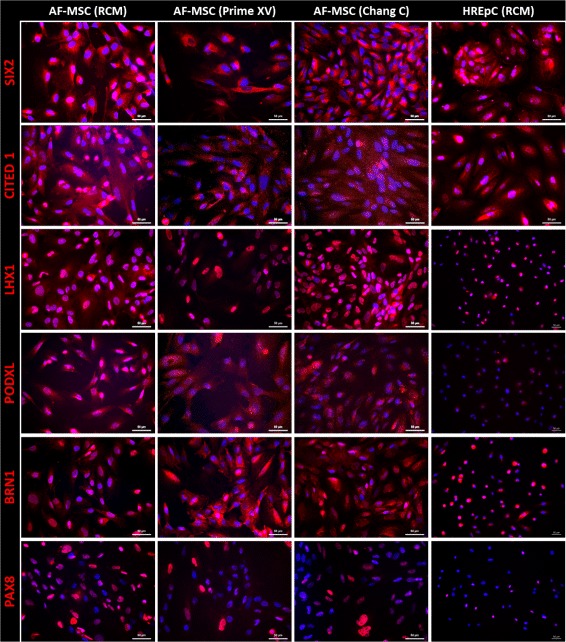
Renal-specific marker expression in AF-MSCs. Immunofluorescent-based images showing expression and localization of SIX2, CITED1, PAX8, BRN1, PODXL and LHX1 in AF-MSCs cultured in either RCM, PrimeXV or Chang C. HREpCs cells served as positive control. Cell nuclei stained using Hoechst/DAPI. AF-MSC amniotic fluid mesenchymal stem cell, HREpC human renal epithelial cell, RCM renal cell medium

To validate our hypothesis that third-trimester AF-MSCs harbor renal progenitor cells, we analyzed AF-MSCs cultured in RCM, which is a medium formulated for kidney cells, for the expression of kidney-associated markers SIX2, CITED1, LHX1, PODXL, BRN1 and Paired-Box-Protein 8 (PAX8)—these were positive. Prime XV and Chang C, specialized for AFC culture, also supported expression of the renal markers. The commercially bought human kidney cells HREpCs served as a positive control. These results imply that AF-MSCs are of nephrogenic origin and the phenotype is maintained irrespective of the media used (Fig. 4).

### AF-MSCs are able to transport albumin

**Fig. 5:**
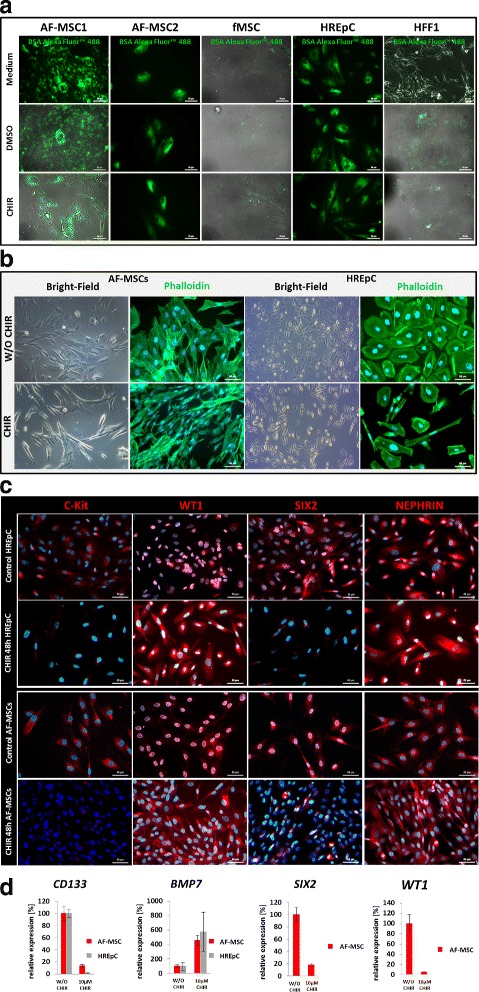
Functional characterization of AF-MSCs as renal cells. Regardless of differentiated or undifferentiated AF-MSC status, functional albumin endocytosis observed at significantly higher levels than in HFF1 and fetal MSCs (fMSC) when cells incubated with albumin at 37 °C (**a**). Activation of WNT signaling by supplementation with GSK3-inhibitor CHIR99021 led to differentiation into tubular looking cells, shown by phalloidin staining (**b**). WT1 localization switch from nucleus to cytoplasm, Nephrin expression retained and C-Kit and SIX2 expression decreased (**c**). Cell nuclei stained using Hoechst/DAPI. qRT-PCR of CHIR differentiated cells clearly showed downregulation of renal undifferentiated progenitor markers *CD133*, *SIX2* and *WT1* and upregulation of the differentiation marker *BMP7* (**d**). AF-MSC amniotic fluid mesenchymal stem cell, DMSO dimethylsulfoxide, fMSC fetal mesenchymal stem cell, HFF human foreskin fibroblast, HREpC human renal epithelial cell, w/o without

**Fig. 6:**
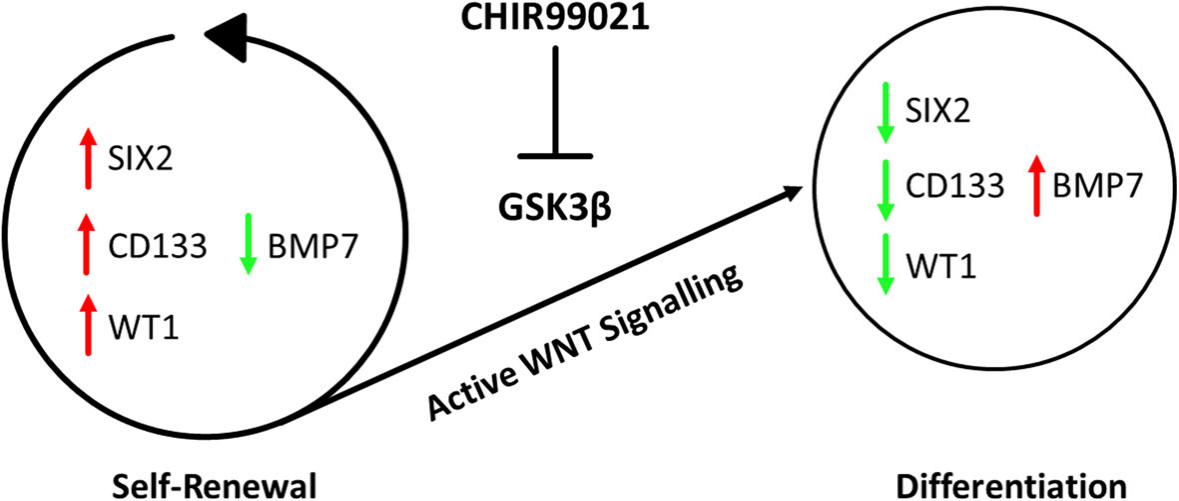
WNT mediated cell fate decisions in AF-MSCs. Self-renewal renal progenitor cells maintained when WNT signaling is inactive due to sustained upregulated expression of *SIX2*, *WT1* and *CD133* and downregulation of *BMP7* on mRNA level. Upon activation of canonical WNT signaling (i.e., CHIR99021-mediated GSK3β inhibition) there is an exit of self-renewal, due to upregulated expression of *BMP7*, but downregulation of *WT1*, *SIX2* and *CD133* expression on mRNA level

Albumin endocytosis is a criterion defining renal cells. To analyze the ability of AF-MSCs to take up and release albumin, fluorescent dye-coupled Alexa Fluor™ 488-labeled albumin was used. It could be shown that the temperature-dependent (37 °C) uptake of albumin (1 h incubation) by AF-MSCs was higher in comparison to fetal MSCs (fMSCs) and human fetal foreskin cell HFF1. This held true for both progenitor and differentiated cells (Fig. 5a). To differentiate AF-MSCs, we treated the cells with 10 μM CHIR99021 (WNT pathway activation by GSK3 inhibition) for 2 days and observed morphological changes from fibroblastic to elongated tubular shape (Fig. 5b), resulting in decreased expression of progenitor markers C-Kit and SIX2. The expression and localization of WT1 switched from nuclear to cytoplasmic upon CHIR99021 treatment whereas Nephrin (NPHS1) expression was stable (Fig. 5c) as detected at the protein level via immunofluorescent-based staining. Real-time RT PCR revealed downregulation of *SIX2*, *WT1* and *CD133* and activation of kidney-associated bone morphogenic protein 7 (*BMP7*) (Fig. 5d). Based on our experimental data we derive a scheme describing CHIR99021-mediated WNT activation and its influence on differentiation or self-renewal (Fig. 6). Self-renewal (inactive WNT signaling) is maintained by elevated expression of the renal progenitor markers *SIX2*, *WT1* and *CD133* (stem cell proliferation marker) and downregulated expression of *BMP7*. In contrast, upon activation of canonical WNT signaling by GSK3β inhibition with CHIR99021, AF-MSCs exit self-renewal and differentiate as a consequence of elevated *BMP7* expression and downregulation of *SIX2*, *WT1* and *CD133* respectively.

### Increased appearance of renal-associated genes in AFCs correlates with gestational time

**Fig. 7:**
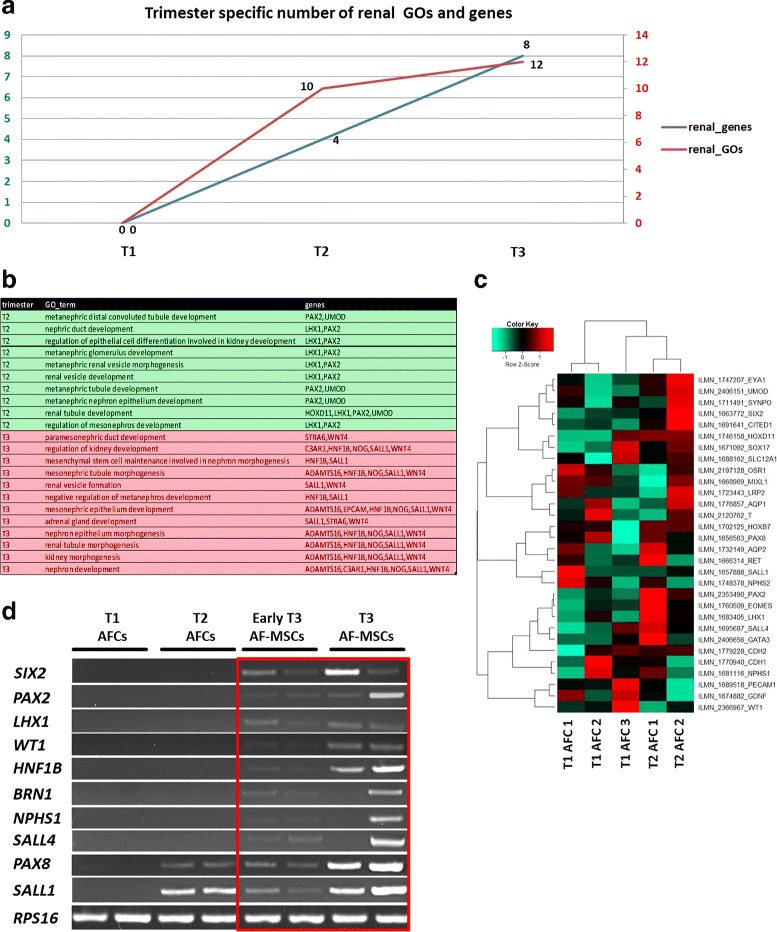
Expression levels of renal-specific genes in AFCs increase with gestational time. Number of renal-enriched genes (blue line) and GOs of trimester-specific AFCs (red line) indicates increasing pattern during fetal kidney development (**a**). Differentially expressed genes involved in nephron development in second and third-trimester AFCs. Gene-set enrichment analysis revealed expression of genes from different renal developmental compartments such as metanephros development (*ADAMTS16*, *EPCAM*, *HNF1B*, *NOG*, *SALL1*, *WNT4*), metanephric mesenchyme development (*HNF1B*, *SALL1*), renal tubule development (*HOXD11*, *LHX1*, *PAX2*, *UMOD*), metanephric nephron and tubule development (*PAX2*, *UMOD*), and metanephric glomerulus and mesonephros development (*LHX1*, *PAX2*) (**b**). Heatmap showing relative expression of genes involved in nephrogenesis from existing published data of AFCs from first and second trimester (**c**). Semi-qPCR of renal genes in AFC/AF-MSC samples from first, second, early third and late third trimester. Gel bands indicating enriched expression of renal genes in third trimester boxed in red (**d**). AFC amniotic fluid cell, AF-MSC amniotic fluid mesenchymal stem cell, GO gene ontology, T1 first trimester, T2 second trimester, T3 third trimester

Taking advantage of previously published transcriptome data [45], related to renal system development from first and second-trimester AFCs, we analyzed gene set enrichments and related GOs from T1 (first trimester), T2 (second trimester) and T3 (third trimester). Interestingly, the number of expressed kidney-associated genes increased with gestational time. From the predictions based on the datasets, we observed that most of the renal developmental-related genes (eight genes) were expressed in the third trimester. Subsequently, only four genes were identified to be present in the second trimester and none was predicted to be expressed in the first trimester. In line with this, no kidney development-related GO terms were found for AFC samples from the first trimester, 10 GOs were shown for the second and 12 GOs in the third trimester, thus implying an increasing renal expression pattern during fetal kidney development which is also shown in a heatmap generated from the transcriptome data (Fig. 7a–c). These data were validated by semi-qPCR of cDNA samples obtained from the mRNA of AFCs/AF-MSCs from the first, second, early third and late third trimesters. We did not detect renal gene expression from the first-trimester samples; in contrast, *PAX8* and *SALL1* could be detected in the second and third-trimester samples. In contrast to this, third-trimester cells exclusively expressed *SIX2*, *PAX2*, *LHX1*, *WT1*, *HNF1B*, *BRN1*, *NPHS1* and *SALL4* (Fig. 7d).

### Transcriptome analysis of third-trimester AF-MSCs reveals involvement in kidney specific biological processes

**Fig. 8:**
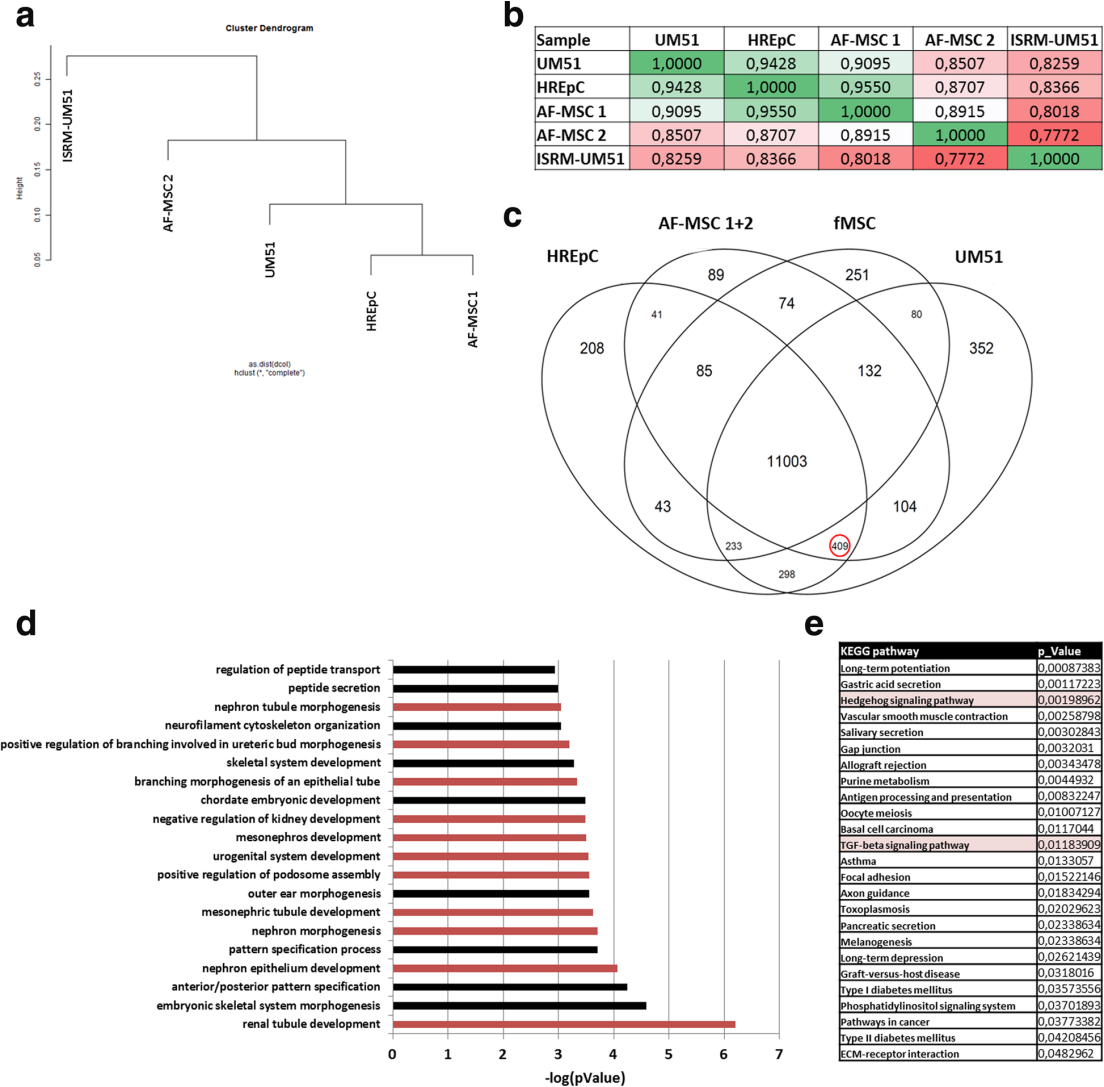
Third-trimester AF-MSCs express genes associated with renal-specific biological processes and have high overlap with kidney cells. Cluster dendrogram shows close relationship of AF-MSCs with UM51 and HREpCs (**a**), confirmed by Pearson correlation coefficients (**b**). Venn diagram analysis extracted 409 genes shared amongst AF-MSCs, UM51 and HREpCs (red circle) (**c**). Top 20 GO terms of the common 409 genes listed depending on their –log(*p* value), renal-related GOs indicated with red bars (**d**). Significant KEGG pathways for the 409 genes. Stem cell-related pathways shaded red (**e**). AF-MSC amniotic fluid mesenchymal stem cell, ECM extracellular matrix, fMSC fetal mesenchymal stem cell, HREpC human renal epithelial cell, KEGG Kyoto Encyclopedia of Genes and Genomes, TGF transforming growth factor

To reveal the AF-MSC identity, the cells’ transcriptomes were compared to HREpCs, UM51 (urine-derived SIX2-positive renal cells) and UM51-derived iPSCs (ISRM-UM51). Using cluster dendrogram analysis, AF-MSCs were shown to cluster together with two different kidney cells and apart from the iPSCs (Fig. 8a). This is also shown by Pearson correlation coefficient calculation (Fig. 8b), revealing a value of 0.9095 for AF-MSC 1 and UM51 and a value of 0.955 for AF-MSC 1 and HREpCs. Next, we wanted to focus on genes shared amongst the AF-MSCs and the other two renal cell types. Since UM51, HREpCs and AF-MSCs showed expression of MSC markers, a sample of bone marrow-derived fetal MSCs was included in the Venn diagram, to allow focus on commonly expressed genes in AF-MSCs, UM51 and HREpCs but not in fMSCs (409 genes) (Fig. 8c). Using these genes, a GO term analysis was conducted. Among the top 20 GOs (Fig. 8d), 11 GOs were connected to kidney development-related biological processes such as “renal tubule development” and “nephron epithelium development”. The significant KEGG pathways resulting from the 409 shared genes are shown in Fig. 8e, revealing stem cell-related pathways such as “TGF-beta signaling pathway” and “Hedgehog signaling pathway”. The complete gene lists, GOs (BP, CC, MF) and the KEGG pathways for each single group as well as for a group consisting of AF-MSCs, UM51 and HREpCs are provided in Additional files 2, 3 and 4.

## Discussion

Amniotic fluid cells display a spectrum of morphologies (Fig. 1a) depicting their composition of fetal-derived differentiated and undifferentiated progenitor cells [24, 33]. In the majority of studies, the heterogeneity of AF-derived cells has led to conflicting results and uncertainty regarding the identity of the cell population, in particular the origin of the third-trimester AFCs [37]. In our earlier work it could be shown that first-trimester AFCs have a germ cell origin [45]. Remarkably, we observed that third-trimester AFCs have similar morphologies when compared to urine-derived cells and human kidney biopsy-derived cells (Fig. 1b), as shown by others [46–50]. It is well known that AF contains various cell types which originate mostly from fetal urine [34, 51] and the appearance of cells in the amniotic fluid/fetal urine increases in number with gestational age. Furthermore, it has also been shown recently that urine cells have a kidney origin [35–37, 40]. In studies on full-term male fetal AF-MSC transcriptomes, we and others found that most of the expressed genes were related to kidney and skeletal system development [32, 37]. Nevertheless, the phenotypes of AFCs obtained during culture were dictated by culture conditions [52] and by the passage number. Initially, we cultured the cells in five distinct media, namely RCM, Prime XV, Chang C, MG30 and αMEM. Regardless of the media used, homogeneous spindle-shaped mesenchymal like cells were observed after a few passages, except for αMEM and MG30 which also led to decreased growth rates. So, these media were excluded from further analysis.

In this study, the third-trimester AFCs were shown to maintain expression of pluripotency-associated stem cell markers C-Kit, SSEA4, TRA-1-60 and TRA-1-81 but not nuclear OCT4 (Fig. 2a), which has also been shown for a subpopulation of urine-derived stem cells [46, 48]. C-kit was also identified to be expressed at the loop of Henley and distal tubules of murine kidney [53]. Second-trimester human nephron progenitor cells were shown to have elevated expression of NANOG and OCT4 [14]. These data suggests that pluripotency-associated gene/protein expression decreases with gestational time.

Third-trimester AFCs investigated in the present work expressed the mesenchymal marker Vimentin, typical MSC cell surface markers CD73, CD90, and CD105 as well as the stem cell marker CD133. Furthermore, the cells differentiated into adipocytes, osteocytes and chondrocytes (Fig. 3a–c), which has also been reported for cells derived from human kidneys [13], and hence we refer to the cells as AF-MSCs. In support of our findings, AFCs as well as cells derived from preterm neonatal urine were described to be positive for Vimentin and CD133 but negative for hematopoietic cell markers [35], which also was confirmed for fetal and adult kidney-derived cells [13, 14, 54]. In addition, our AF-MSCs also express CK19 (Fig. 3a) as shown previously for human kidney cells [53]. It has been described that subpopulations of renal cells exist with MSC-specific morphology and marker expression (CD73, CD105) which additionally express metanephric mesenchyme markers such as SIX2, CITED1 and PAX2 [37, 54].

As the AF-MSCs share similar properties with neonatal urine cells and human renal cells with respect to pluripotency-associated and mesenchymal marker expression, and multipotent differentiation potential, we assumed, as shown in the 1970s [51], that AFCs originate from fetal kidney. This hypothesis is supported by a recent review describing that cells leave the fetal kidney during the transition from the pronephron to the metanephron, and reside within AF [37]. To confirm, we investigated AF-MSCs cultured in RCM, Chang C and Prime XV for the expression of typical renal markers such as SIX2, CITED1, PODXL, LHX1, BRN1 and PAX8. Irrespective of the used media, the third-trimester AF-MSCs expressed all of these markers (Fig. 4). In line with our results, nephron progenitor cells derived from the developing human kidney as well as from neonatal urine were previously reported to express the investigated marker [14, 15, 35, 55, 56].

We also investigated uptake of exogenous albumin as a key kidney function, which has been shown for human renal cells [57–59] as well neonatal urine cells [35]. Third-trimester AF-MSCs and HREpCs showed albumin endocytosis whereas fMSCs and HFF1 did not (Fig. 5a). Further, we analyzed the changes in morphology and protein/gene expression in the AF-MSCs upon differentiation by activation of WNT signaling as a consequence of inhibition of GSK3 using CHIR99201. Cell shapes became elongated and expression of renal progenitor markers C-KIT and SIX2 decreased, WT1 expression translocated from the nucleus to the cytoplasm and Nephrin expression remained cytoplasmic, which was also confirmed for cells isolated directly from a human kidney biopsy [37, 60]. The translocation of WT1 expression from the nucleus to cytoplasm has been described previously [61]. The cytoplasmic expression of WT1 will denote a loss of transcription factor activity.

Moreover, qRT-PCR revealed that the differentiated AF-MSCs acquired upregulated *BMP7* expression with parallel decreased *SIX2*, *WT1* and *CD133* expression (Fig. 5d), as shown previously [62, 63]. One possible explanation for our observations could be that the antibody recognizes an epitope present on a variant of WT1 which we could not detect with our primers. Of course this is speculation and more studies are required to substantiate this observation.

Since nephrons are generated during the second and third trimesters [64], a synergistic relation between expression of kidney-associated genes in AF-MSCs and the gestational period can be postulated. To address this, we analyzed gene expression from previously published transcriptome data [32, 45] and performed a semiquantitative PCR of first, second and third-trimester samples to investigate any possible relationship between gradual expressions of renal genes with gestational time. The third-trimester AF-MSCs expressed increasing number of genes and GOs compared to second-trimester cells (Fig. 7a, b, d). For future research it would be of high value to have AF samples obtained from gestational week-wise time points to better understand the impact of the temporal developmental stage of the fetal kidney on the composition of the AF and likewise the identity of AF-MSCs. Cluster dendrogram analysis based on the transcriptome data showed that AF-MSCs cluster apart from pluripotent cells and cluster together with kidney-derived cells (Fig. 8a, b). In line with this, AF-MSCs shared more genes with human kidney cells UM51 and HREpCs than with fetal MSCs (Fig. 8c). Furthermore, GO analysis focusing on biological processes revealed the involvement of genes associated mostly with kidney development (Fig. 8d), which also can be observed in our previous analysis of AF specific genes [32].

Since the kidney is a complex organ and is composed of a multitude of different cell types, sorting and specification of distinct kidney cells from the amniotic fluid needs to be investigated.

Nevertheless, our results identify third-trimester human amniotic fluid-derived mesenchymal stem cells as of renal origin. As we have demonstrated before that these AF-MSCs also secrete immunomodulatory factors, they are highly suitable for transplantation, for example in chronic/acute kidney disease or graft versus host disease. These findings qualify these cells and the iPSCs derived from them as potent cells that can be used in the future for research on nephrogenesis, for modeling kidney-related diseases and for drug screening in combination with tissue engineering approaches such 3D organoid formation to further improve mimicking of kidney features in vitro.

## Conclusions

We have demonstrated that third-trimester human AFCs originated from fetal kidney are mesenchymal stem cells (AF-MSCs) with retained renal cell gene expression and functionality. AFCs/AF-MSCs have been widely investigated; however, to date only a limited number of studies have attempted to reveal their enigmatic origin. Our results add an important milestone for the usefulness of these cells as a suitable source for future studies related to nephrogenesis, derivation of iPSCs, nephrotoxicity tests and kidney disease-related cell replacement therapies.

## Additional files

Additional file 1: **Table S1.** Antibodies used in this study **Table S2.** Primers used for semiquantitative and quantitative real-time PCR. (DOCX 18 kb)

Additional file 2: Gene list of exclusive groups and shared genes of AF-MSCs, HREpCs and UM51. (XLS 995 kb)

Additional file 3: GOs of exclusive groups and shared genes of AF-MSCs, HREpCs and UM51. (XLS 331 kb)

Additional file 4: KEGG pathways of exclusive groups and shared genes of AF-MSCs, HREpCs and UM51. (XLSX 76 kb)

## Abbreviations

AF: Amniotic fluid
AFC: Amniotic fluid cell
AF-MSC: Amniotic fluid mesenchymal stem cell
BM-MSC: Bone marrow-derived mesenchymal stem cell
BSA: Bovine serum albumin
CD: Cluster of differentiation
DAVID: Database for Annotation, Visualization and Integrated Discovery
ESC: Embryonic stem cell
FBS: Fetal bovine serum
fMSC: Fetal mesenchymal stem cell
GO: Gene ontology
HFF: Human foreskin fibroblast
HREpC: Human renal epithelial cell
iPSC: Induced pluripotent stem cell
KEGG: Kyoto Encyclopedia of Genes and Genomes
αMEM: Minimum essential medium alpha modification
MSC: Mesenchymal stem cell
PBS: Phosphate buffered saline
PCR: Polymerase chain reaction
RCM: Renal cell medium
REGM: Renal epithelial growth medium
RT: Room temperature
T1: First trimester
T2: Second trimester
T3: Third trimester
