# Supplementary material for: The presence of human mesenchymal stem cells of renal origin in amniotic fluid increases with gestational time
Source: Stem Cell Res Ther. 2018 Apr 25;9:113. doi: 10.1186/s13287-018-0864-7 (PMC5918774; doi:10.1186/s13287-018-0864-7)
Supplement: Supplementary file 1 — Table S1. Antibodies used in this study Table S2. Primers used for semiquantitative and quantitative real-time PCR. (DOCX 18 kb) [file 13287_2018_864_MOESM1_ESM.docx]

**Additional file 1**

Table S1: List of Antibodies used in this study

| Primary Antibodies | Providing company | Dilution ratios |
| --- | --- | --- |
| OCT-4A (C30A3) rabbit mAb number 2840 | Cell Signaling Technology, USA | IF 1:400 |
| SSEA4 (MC813) mouse mAb number 4755 |  | IF 1:1000 |
| E-cadherin (24E10) rabbit mAb number 3195 |  | IF 1:200 |
| Vimentin (5G3F10) mouse mAb number 3390 |  | IF 1:200 |
| TRA-1-60 mouse mAb number 4746 |  | IF 1:1000 |
| TRA-1-81 mouse mAb number 4745 |  | IF 1:1000 |
| CD133 PA2049 | Boster Bio, USA | IF 1:500 |
| Rabbit anti-CK19, NB100-687 | Novus Biologicals | IF 1:100 |
| C-Kit (H-300) rabbit polyclonal IgG | Tebu Bio, Germany | IF 1:200 |
| rb SIX2 Poly AP, 11562-1-AP | Proteintech | IF 1:200 |
| CITED1, PA5-40585 | Invitrogen | IF 1:160 |
| rb POU3F3 Poly AP /Brn1, 18999-1-AP | Proteintech Euro | IF 1:200 |
| PAX8 (D2S2I) rabbit mAb, 59019s | Cell Signaling Technology, USA | IF 1:200 |
| rb Anti WT1 clone 6F-H2, O5-753 | MD Millipore Corp. | IF 1:200 |
| rb Nephrin AB, PA5-20330 | Invitrogen | IF 1:200 |
| rb PODXL, sc33138 | Santa Cruz Biotech. | IF 1:200 |
| rb LIM1/LHX1, ab14554 | abcam | IF 1:500 |

Table S2: List of Primers used for semi-qRT-PCR and qRT-PCR

| Genes Name | Primer Sequences | Product size (bp) |
| --- | --- | --- |
| *SIX2* | F1: GGCCAAGGAAAGGGAGAACA, R1: AGCTGCCTAACACCGACTTG | 86 |
| *PAX8* | F1: CCCCCTACTCCTCCTACAGC, R1: ACTGTCCCCATGGCAACTAC | 147 |
| *WT1* | F1: CCAAAGGAGACATACAGGTGTGA, R1: GTCCTGGTGTGGGTCTTCAG | 93 |
| *PAX2* | F1: ACTCCATCAATGGGATCCTG, R1: CCACACCACTCTGGGAATCT | 111 |
| *LHX1* | F1: TTCTCCAGGGAAGGCAAACTC, R1: CCAGGTCGCTAGGGGAGAT | 100 |
| *BRN1* | F1: CACTTCCTCAAGTGCCCCAA, R1: GCGAGTAGACGTCGTCGG | 169 |
| *SALL1* | F1: TCTAGGAGGCAATCCCGTCA, R1: GCGCTGCTGCATACTGATTC | 107 |
| *SALL4* | F1: ACCCCAGCACATCAACTCG, R1: CATTCCCTGGGTGGTTCACT | 134 |
| *RPS16* | F1: GCTATCCGTCAGTCCATCTCCAA, R1: CCTTCTTGGAAGCCTCATCCAC | 73 |
| *HNF1B* | F1: CGCTCTGTACACCTGGTACG, R1: ACAGCAGCTGATCCTGACTG | 133 |
| *NPHS1* | F1: GTGCACTATGCTCCCACCAT, R1: ATTGGCATCGACAGTGCAGA | 93 |
| *BMP7* | F1: CAACCTCGTGGAACATGACAAG, R1: AAGATCAAACCGGAACTCTCGAT | 70 |
| *CD133* | F1: GACTTGCGAACTCTCTTGAATGA, R1: GGTAGTGTTGTACTGGGCCAAT | 222 |
| *RPL37A* | F1: GTGGTTCCTGCATGAAGACAGTG, R1: TTCTGATGGCGGACTTTACCG | 84 |
